# Surveillance for West Nile Virus in American White Pelicans, Montana, USA, 2006–2007

**DOI:** 10.3201/eid1603.090559

**Published:** 2010-03

**Authors:** Gregory Johnson, Nicole Nemeth, Kristina Hale, Nicole Lindsey, Nicholas Panella, Nicholas Komar

**Affiliations:** Montana State University, Bozeman, Montana, USA (G. Johnson, K. Hale); Centers for Disease Control and Prevention, Fort Collins, Colorado, USA (N. Nemeth, N. Lindsey, N. Panella, N. Komar)

**Keywords:** West Nile virus, zoonoses, American white pelicans, Pelecanus erythrorhynchos, surveillance, epidemiology, viruses, Montana, research

## Abstract

West Nile virus (WNV)–associated deaths of American white pelican (*Pelecanus erythrorhynchos*) chicks have been recognized at various nesting colonies in the United States since 2002. We evaluated American white pelican nesting colonies in Sheridan County, Montana, USA, for an association between WNV-positive pelican carcasses and human West Nile neuroinvasive disease. Persons in counties hosting affected colonies had a 5× higher risk for disease than those in counties with unaffected colonies. We also investigated WNV infection and blood meal source among mosquitoes and pelican tissue type for greatest WNV detection efficacy in carcasses. WNV-infected *Culex tarsalis* mosquitoes were detected and blood-engorged *Cx. tarsalis* contained pelican DNA. Viral loads and detection consistency among pelican tissues were greatest in feather pulp, brain, heart, and skin. Given the risks posed to wildlife and human health, coordinated efforts among wildlife and public health authorities to monitor these pelican colonies for WNV activity are potentially useful.

## Abstract 2

Wildlife disease investigations may help reduce zoonotic infections.

After West Nile virus (WNV; family *Flaviviridae*, genus *Flavivirus*) was detected in the Great Plains of the United States in 2002, programs were initiated to identify the spatial distribution of WNV transmission risk throughout the region. Surveillance activities included compiling case counts for human and equine disease, and testing mosquitoes, avian carcasses, and sentinel chicken serum samples for WNV infection. Corvid (primarily crows and magpies) death surveillance was an effective early warning system for human disease shortly after WNV was detected in this region (*1*). However, carcasses of numerous other bird species also were positive for WNV (*2*). Avian deaths caused by WNV infection typically result in widely dispersed carcasses; for the extent of these deaths to be recognized, substantial public cooperation is required in reporting deaths (*3*). In contrast to this cryptic pattern of deaths, geographically focused deaths among juvenile American white pelicans (*Pelecanus erythrorhynchos* Gmelin; order Pelecaniformes, family Pelecanidae) have occurred as a result of WNV transmission at numerous pelican-breeding colonies throughout the northern Great Plains (*4*). This region of the United States has the highest incidence of human West Nile neuroinvasive disease (WNND) recorded (*5*).

Concurrent with the arrival of WNV to the northern Great Plains region, high death rates of pelican chicks were observed at 4 major colonies in Montana, North Dakota, South Dakota, and Minnesota. WNV was presumed to be the etiologic agent for >9,000 American white pelican deaths in 7 states in 2002–2003 on the basis of testing of a sample of carcasses from various affected colonies (*6*). At Medicine Lake National Wildlife Refuge (MLNWR) in Montana, the chick death rate from mid-July until fledging, a time when pelican chicks are less vulnerable to severe weather and predation, typically averages <4%. However, this death rate reached as high as 44% among colonies in the region after the arrival of WNV in 2002, and annual losses since then have remained elevated (typically 7–8×) in most years (*4*). Although a spatiotemporal link between WNV detection and pelican chick deaths seems evident, the cause of most of these deaths remains presumptive. Furthermore, the potential public health consequences of American white pelican deaths need to be evaluated.

Pelican deaths may indicate increased risk for WNV transmission to persons living in nearby communities. We evaluated pelican deaths and human WNND cases for potential associations. In addition, we captured and tested mosquitoes from MLNWR in 2006 and 2007 to determine the risk for vector-borne transmission of WNV and identify the vertebrate source of mosquito blood meals. Finally, we collected a series of tissue types from a subset of pelican carcasses at our field site to identify the most efficient tissue for maximizing the probability of WNV detection and to confirm WNV infection as a contributing factor to elevated prefledgling pelican death rates.

## Materials and Methods

### Site Description

MLNWR (elevation 590 m) is located in Sheridan County in northeastern Montana (48°27′N, 104°23′W). The refuge covers 13,000 hectares, including Medicine Lake (3,320 hectares), the largest natural lake in eastern Montana. Extensive wetlands provide suitable breeding habitat for mosquitoes and aquatic birds. Cropland and short-grass prairie surround the lake and provide nesting grounds to ≈125 species of birds. Approximately 4,000 breeding pairs of pelicans nest on a narrow peninsula (length ≈500 m) (*7*).

### Spatiotemporal Associations between Pelican Deaths and Human WNV Disease

Data for human WNV disease cases were obtained from ArboNET, an internet-based passive surveillance system maintained by the Centers for Disease Control and Prevention (Fort Collins, CO, USA) in collaboration with state and local health departments. Locations of colonies were obtained from King and Anderson (*8*). Wildlife Mortality Quarterly Reports published by the US Geological Survey National Wildlife Health Center provided locations and dates of WNV-related pelican deaths during 2003–2007 (www.nwhc.usgs.gov/publications/quarterly_reports/index.jsp). We used an odds ratio to compare incidence of WNND cases in counties with reported WNV-associated deaths at pelican-nesting colonies to that in all other counties with pelican colonies during 2003–2007. Positive and negative predictive values were defined as the percentage of counties with pelican-nesting colonies in which a pelican WNV die-off and human WNND case(s) occurred, and in which neither a pelican WNV die-off nor a human WNND case occurred, respectively, during a given year.

Although neuroinvasive and nonneuroinvasive disease cases are reportable, reporting of nonneuroinvasive WNV disease has varied substantially by jurisdiction and over time. Therefore, only WNND cases were considered. For each year and county that pelican and human WNV disease were observed, we determined the interval between the earliest collection date of WNV-positive pelicans and the earliest onset date of human WNV disease.

### Surveillance of Mosquitoes

Mosquitoes were collected from MLNWR by using battery-powered miniature light traps (J.W. Hock, Gainesville, FL, USA) supplemented with CO_2_ from a 9-kg compressed gas tank. In 2006, seven traps were placed at 5 locations on the northeast perimeter of Medicine Lake; 3 were at Bridgerman Point, 10–200 m from pelican-nesting and -congregation sites (*9*). In 2007, all 5 traps were placed at Bridgerman Point. Traps were generally operated for 2 consecutive nights each week from mid-May through August in 2006 and 2007, and collections were stored at –20°C for >24 h before transport on dry ice. Collections were processed on a chill table and the light trap index (LTI) was calculated for each week as the number of trapped *Culex tarsalis* mosquitoes per trap night.

For WNV testing by reverse transcription–PCR (RT-PCR), weekly trap collections were sorted by species and location. Pools of <50 adult female mosquitoes were homogenized in vials containing 4.5 mm-diameter copper-coated steel beads (BB pellets) in BA-1 medium (medium 199 with Hanks balanced salt solution, 0.05 mol/L Tris buffer, pH 7.6, 1% bovine serum albumin, 0.35 g/L of NaHCO3, 100 mg/L streptomycin, 100 U/mL penicillin G, 1 μg/mL amphotericin B) and clarified by centrifugation. RNA was extracted from the supernatant and purified through an EasyMag extractor (bioMérieux, Durham, NC, USA) by using automated magnetic silicon extraction. Purified RNA was transcribed into cDNA and amplified by using specific WNV primers as described (*10*) in an EasyQ thermocycler (bioMérieux). Detection of WNV cDNA was achieved by agarose gel electrophoresis. Prevalence of WNV infection in mosquito populations was estimated by using PooledInfRate (www.cdc.gov/ncidod/dvbid/westnile/software.html). Vector index was calculated as the product of the LTI and infection rate (*11*).

### Identification of Mosquito Blood Meals

Blood-engorged *Aedes vexans* and *Cx*. *tarsalis* mosquitoes were stored individually at –20°C and processed for blood meal identification as described (*12*). Briefly, each mosquito was homogenized and DNA was extracted. A portion of the mitochondrial cytochrome B gene was amplified and sequenced, and the resulting sequence was compared with sequences in a database for species identification.

### Pelican Sample Collection and Preparation

Moribund pelican chicks (≈6–12 weeks of age) showing clinical signs suggestive of WNV infection (e.g., ataxia, torticollis, reluctance or inability to move) (Figure) were killed by cervical dislocation and stored frozen until necropsy. In 2006, carcasses were collected from July 25 through August 11 (n = 8) during a period of maximum chick deaths, and in 2007 from July 11 through August 1 (n = 24) after confirmation of WNV in mosquito pools. Also in 2007, oropharyngeal and cloacal swab samples, skin, and feather samples were collected from 23 carcasses in the field before they were frozen for comparison with samples collected from the same animals in the laboratory during necropsy.

**Figure:**
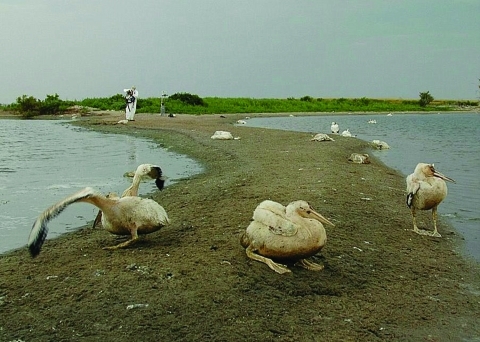
Juvenile American white pelicans (*Pelecanus erythrorhynchos*) at Medicine Lake National Wildlife Refuge, Montana, USA, 2007, including ill (foreground) and dead (background) birds.

For swab samples, dacron-tipped applicators were inserted into the oropharyngeal cavity (behind the pouch) or into the cloaca and then submerged and swirled in vials containing 1 mL BA-1 medium and discarded. In 2007, eye swab samples were collected from 17 carcasses by placing the applicator tip between the inner membrane of the eyelid and the eye. In addition, pouch lice (*Piagetiella peralis*) were individually removed from the inner lining of the pouch of each pelican and pooled in cryovials (<40 lice/pool). Four flight feathers were removed from each carcass (2/wing). Feathers were removed from the follicle, and the calamus (quill tip) was aseptically cut and placed with the associated pulp into a vial containing 1 mL BA-1 medium. Approximately 0.5 cm^3^ each of skin, kidney, spleen, heart, lung, and brain was aseptically collected and placed in cryovials containing 1 mL BA-1 medium for cryopreservation.

Tissues and chewing lice, obtained from the inside of throat pouches, were homogenized in a mixer mill (5 min at 25 cycles/s; Retsch GmbH, Haan, Germany) in 1 mL of BA-1 medium containing 20% fetal bovine serum and a BB pellet. Homogenates were clarified by centrifugation (12,000 × *g* for 3 min), and an aliquot was removed for immediate testing. Remaining supernatants were stored at –80°C.

### Detection of WNV in Pelican Samples

Virus isolation was performed for tissues, lice homogenates, and swabs by using a Vero cell plaque assay as described (*13*). Viral plaques were confirmed as WNV by RT-PCR or VecTest WNV Antigen Detection Assay (Medical Analysis Systems, Camarillo, CA, USA) as described (*2*). RT-PCR and plaque assay detection methods were compared within tissue types by using the Fisher exact test with Bonferroni correction for 9 comparisons (α = 0.0056). For specimens collected in the field and their carcass-matched controls collected in the laboratory, test results were compared by using the κ statistic for concordance.

RT-PCR methods for detection of WNV RNA in tissues were according to those of Lanciotti et al. (*14*), except for use of the Viral RNA Minikit (QIAGEN, Valencia, CA, USA) for RNA extraction and the Bio-Rad Icycler IQ Real-time Detection System (Bio-Rad, Hercules, CA, USA) for cDNA amplification. A cycle threshold value <37 was considered positive for target sequence amplification. Samples were screened with 1 pair of primers (genome positions were 10668 for forward primer, 10770 for reverse primer, and 10691 for probe) and positive results were confirmed with a second pair of primers (genome positions were 1160 for forward primer, 1229 for reverse primer, and 1186 for probes).

## Results

### Association of Pelican Deaths and Human WNV Disease

The probability of human WNND cases in counties with pelican nesting colonies increased 5× when WNV-associated deaths occurred among the pelicans (odds ratio 5.0, 95% confidence interval 1.9–13.0, n = 135 county-years). The positive predictive value of pelican deaths for human WNND cases was 55%, and negative predictive value was 81%. Pelican deaths were observed an average of 23.1 days (median 13.5 days) before human case onset and occurred before human disease onset in 12 (75%) of 16 county-years (Table 1).

**Table 1:** Deaths in pelicans infected by WNV and WNV human disease in counties with nesting American white pelican colonies, United States, 2003–2007*

Year	County, state	Earliest date of pelican death	Earliest date of disease in humans	Difference, d
2003	Big Stone, MN	Jul 1	Aug 25	56
	Phillips, MT	Aug 1	Aug 15	14
	Sheridan, MT	Jul 23	Aug 1	9
	Stutsman, ND	Jul 15	Jul 26	11
	Day, SD	Jul 28	Jul 14	−14
2004	Big Stone, MN	May 30	Sep 1	93
2005	Sheridan, MT	Jun 23	Jul 14	52
	Stutsman, ND	Jun 17	Jul 18	31
	Day, SD	Jul 6	Aug 26	51
2006	Big Stone, MN	Jun 15	Jul 27	42
	Washoe, NV	Jul 14	Jul 10	−4
	Stutsman, ND	Jul 24	Aug 1	8
	Day, SD	Jul 19	Aug 1	13
	Brown, WI	Jul 15	Aug 10	26
2007	Stutsman, ND	Jul 7	Jul 1	−6
	Day, SD	Jul 9	Jun 27	−12

*WNV, West Nile virus.

### Mosquito Surveillance

In 2006, a total of 414 *Cx*. *tarsalis* mosquitoes were captured in 67 light trap-nights from July 1 through August 5. Weekly LTI values for *Cx*. *tarsalis* mosquitoes ranged from 4.1 to 9.0 during July and early August (Table 2) when mosquito populations were low because of severe drought (larval production sites were dry or contained ephemeral water). None of 12 pools of *Cx*. *tarsalis* mosquitoes assayed were positive for WNV RNA.

**Table 2:** *Culex tarsalis* mosquito infection data for WNV calculated weekly during 2 WNV transmission seasons, Medicine Lake National Wildlife Refuge, Montana, USA*

Week of collection	2006				2007				
	Light trap nights	Light trap index ± SD†	No. positive pools/no. tested		Light trap nights	Light trap index ± SD†	No. positive pools/no. tested	Infection rate‡ (95% CI)	Vector index§ (95% CI)
Jul 1–7	13	4.1 ± 4.3	0/17		4	448.5 ± 549.5	0/100	0.0	0.0
Jul 8–14	9	6.6 ± 11.6	0/59		9	325.3 ± 131.4	5/800	6.3 (0.8–11.7)	2.0 (0.3–3.8)
Jul 15–21	10	9.0 ± 8.8	0/88		10	1,643.0 ± 899.8	7/1,000	7.0 (1.8–12.2)	11.5 (3.0–20.0)
Jul 22–28	11	7.0 ± 5.9	0/69		8	259.6 ± 301.9	4/1,000	4.0 (0.1–7.9)	1.0 (0.03–2.1)
Jul 29–Aug 4	13	7.1 ± 10.4	0/74		7	169.0 ± 105.3	10/937	10.7 (4.1–17.3)	1.8 (0.7–2.9)

*WNV, West Nile virus: CI, confidence interval. †Light trap index is mean number of adult female *Cx. tarsalis* mosquitoes collected per trap night. Seven traps were used in 2006, and 5 traps were used in 2007. ‡Infection rate is in units of 1,000 mosquitoes and determined by maximum-likelihood estimate. Infection rate was 0 for all collection dates in 2006. §Vector index is the product of light trap index and infection rate. Unit of measure is number of infected female *Cx. tarsalis* mosquitoes per trap night. Vector index values were 0 for all collection weeks in 2006.

In 2007, a total of 25,291 *Cx*. *tarsalis* mosquitoes were captured in 42 light trap-nights from June 30 through August 8 (Table 2). Weekly LTI values during this period ranged from 169 to 1,643. WNV was detected in 28 (32.2%) of 87 mosquito pools by RT-PCR, with the first positive mosquito samples collected during the week of July 8, 2007. WNV was detected in 10 of 20 pools of mosquitoes collected during the last week of July; we observed an estimated infection rate of 10.7/1,000 mosquitoes.

Vertebrate DNA sequences were obtained from blood-engorged abdomens of 22 mosquitoes collected in 2007, including 8 *Ae*. *vexans* and 14 *Cx*. *tarsalis*. All 22 mosquitoes had fed on American white pelicans.

### WNV in Pelican Samples

Twenty-seven (84.4%) of 32 pelicans sampled had >1 tissues positive for WNV by plaque assay compared with 7 (87.5%) of 8 positive by RT-PCR (Table 3). Pelicans with WNV-positive tissues were collected from July 25 through August 11, 2006, and July 11 through August 1, 2007. Because pelicans were not collected before these dates, the timing of initial onset of WNV outbreaks in pelicans is unknown.

**Table 3:** WNV detected by plaque assay or RT-PCR in tissues from American white pelican carcasses collected at Medicine Lake National Wildlife Refuge, Montana, USA, 2006–2007*

Specimen	No. (%) plaque assay positive, n = 27	Median viral titer, log PFU/0.5 cm^3^ (range)†	No. (%) RT-PCR positive, n = 8
Feather pulp	18 (66.7)	3.5 (1.3–5.9)	6 (75.0)
Kidney	18 (66.7)	2.6 (2.0–3.9)	3 (37.5)
Spleen	4 (14.8)	1.6 (0.7–2.0)	1 (12.5)
Brain	21 (77.8)	2.7 (1.7–5.9)	5 (62.5)
Heart	14 (51.9)	3.6 (0.7–5.3)	2 (25.0)
Lung	6 (22.2)	2.4 (1.7–3.3)	1 (12.5)
Skin	25 (92.6)	3.1 (0.7–5.0)	5 (62.5)
Oral swab	9 (33.3)	2.1 (0.7–3.7)	3 (37.5)
Cloacal swab	7 (25.9)	1.8 (0.7–3.7)	2 (25.0)
Eye swab‡	2 (11.8)	2.2 (1.7–2.6)	–

*WNV, West Nile virus; RT-PCR, reverse transcription–PCR. All carcasses were WNV positive by virus isolation or detection of specific WNV RNA by RT-PCR. †Median titers were calculated from the positive specimens only. Unit of measurement for swabs is per swab rather than per 0.5 cm^3^. ‡Eye swabs were collected from only 17 WNV-positive chicks.

Skin was the most efficacious tissue for WNV detection in pelican carcasses. Viral loads were greatest in feather pulp, brain, heart, and skin. RT-PCR and plaque assay results were similar; detection rates did not differ among specific tissues or between field-collected vs. laboratory-derived samples (Table 4). Concordance (i.e., test agreement) was 82% (κ = 0.82) among matched field and laboratory samples from the same carcasses. All pouch lice samples were negative for WNV.

**Table 4:** Virus titers of field-collected samples from WNV-positive American white pelican chicks and test agreement with carcass-matched specimens, Montana, USA, 2006–2007*

Sample	No. (%) WNV positive, n = 19	Median viral titer, log PFU/0.5 cm^3^ (range)	κ
Skin†	15 (88.2)	3.6 (1.7–5.0)	0.88
Feather pulp	15 (78.9)	4.9 (1.3–5.6)	0.84
Oral swab	7 (36.8)	1.2 (0.7–2.1)	0.79
Cloacal swab	4 (21.1)	1.5 (0.7–2.7)	0.79

*WNV, West Nile virus; κ, concordance. All carcasses were positive for WNV infection by virus isolation or detection of specific WNV RNA by reverse transcription–PCR. Only plaque assay results are presented for field-collected samples. †Skin was collected from only 17 carcasses in the field.

## Discussion

We observed an association between human cases of WNND and WNV-induced juvenile pelican deaths in counties with pelican-nesting colonies. The positive and negative predictive values of pelican WNV-associated deaths for human WNND cases were similar in magnitude to those of American crow (*Corvus brachyrhynchos*) deaths. These findings suggest that monitoring of pelican deaths in colonies near human populations could be of potential use in public health–oriented WNV surveillance programs, many of which use crow deaths as indicators of local WNV activity and human risk (*15*).

Surveillance of pelican colonies for WNV activity could assist in presaging human WNV infection and associated disease. Pelican deaths were generally detected >2 weeks before WNV disease onset in humans. However, our observations were limited by numerous assumptions inherent to surveillance data, such as that human case-patients were infected in their home counties, that all human residents of each county were equally at risk for WNV infection, and that only residents of a county with a colony were potentially at risk. Because human settlements nearest a colony may pertain to a different county, a more accurate analysis would evaluate distance from the nesting colony as a risk factor for human cases, independent of county lines.

Most pelican colonies are remote from human population centers and are not currently actively monitored for WNV-associated deaths. Although human population densities near pelican colonies are low, infected host-seeking mosquitoes may travel >10 km in search of a blood meal, especially because breeding pelicans and other birds disperse from the region, typically in August. *Cx*. *tarsalis* mosquitoes have traveled distances <12.6 km (*16*). Other mosquito species, such as *Ae*. *vexans*, could serve as bridge vectors between infectious juvenile pelicans and susceptible humans. Blood meal analyses from engorged *Ae*. *vexans* and *Cx*. *tarsalis* mosquitoes showed that these well-known biters of humans also feed on pelicans. Furthermore, dispersing pelicans may be infectious and introduce the virus to competent mosquitoes near human population centers. Pelicans forage daily <80 km from colony sites (*17*).

We confirmed vector-borne transmission of WNV to pelicans at MLNWR in Sheridan County, Montana. *Cx*. *tarsalis* mosquitoes appeared to be the major vector for transmission of WNV to pelicans in 2007 because vector and trap indices were high for this mosquito species, and blood meal identification linked these vectors to the pelicans. WNV was detected in pelican carcasses in 2006 despite low populations of *Cx*. *tarsalis* mosquitoes and lack of WNV detection in mosquitoes, which suggested that pelican deaths may be a sensitive indicator of local WNV activity. Juvenile pelican deaths caused by WNV infection have been observed (*4*,*6*) but never targeted for WNV avian mortality surveillance, i.e., to generate public health–related data to be used for WNV prevention and control. In the reports of juvenile pelican deaths, comparison of WNV tissue loads in pelicans was not rigorously evaluated.

Biologic specimens collected from avian carcasses have proven useful in WNV surveillance; the American crow has been a useful sentinel (*2*,*3*,*18*). Oral swabs and feather pulp are preferred target samples for diagnosis of WNV infection in corvids (*19*,*20*). To help guide future WNV surveillance efforts, we sought to determine which pelican samples would be most useful for WNV detection. Our results showed that skin and feather pulp are the most ideal specimens. Previous research showed that feather pulp was slightly more efficacious than oral swabs for detecting WNV when the VecTest assay was used for corvids (*21*), and that 100% of feather pulp samples were positive among WNV-infected American crows and blue jays (*Cyanocitta cristata*) (*20*). Feather pulp and skin meet criteria for a low-resource approach to dead bird surveillance: samples require a minimal amount of time for field collection, dissection of the carcass is not required, exposure of laboratory personnel to infected carcasses is avoided, samples can be easily transported and shipped, and laboratory processing costs can be kept to a minimum. Oropharyngeal, cloacal, and eye swab samples were relatively insensitive for detecting WNV in pelicans.

Continued surveillance of American white pelican colonies is useful for assessing long-term effects of WNV in colonies and in populations in the northern plains and upper Midwest region of the United States. The role of these colonial nesting birds in WNV ecology, and conversely that of WNV on pelican ecology, remains unknown. WNV-amplifying hosts and vectors are generally plentiful at pelican colonies, and recurring chick deaths since 2003 suggest that WNV-induced reductions in pelican populations will continue. Because juvenile pelicans are likely more susceptible to WNV-associated illness and death than adults, the effects of WNV on pelican population growth would manifest as failed recruitment of new birds into the population in affected colonies, rather than loss of fertile adults. Indirect environmental effects of pelican nest failures are unknown.

The association we observed between WNV disease among pelicans and humans does not imply that pelicans are the source of human WNV infections, or vice versa, and may merely be a consequence of geographic autocorrelation. However, this link highlights the benefit of communication between wildlife and public health sectors. Knowledge of WNV infections in either sector may signal a problem requiring attention in the other. This study of deaths in pelicans caused by WNV serves as a reminder that wildlife disease investigations may play an useful role in mitigating risk for zoonotic infections in humans.
